# Chronic mercury poisoning: Report of two siblings

**DOI:** 10.4103/0019-5278.64610

**Published:** 2010

**Authors:** Cahide Yilmaz, Mesut Okur, Hadi Geylani, Hüseyin Çaksen, Oğuz Tuncer, Bülent Ataş

**Affiliations:** Department of Pediatric Neurology, Mustafa Kemal University Faculty of Medicine, Hatay, Turkey; 1 Department of Pediatrics, Women's and Children's Hospital, Van, Turkey; 2 Department of Pediatrics , Yüzüncü Yil University Faculty of Medicine, Van, Turkey; 3 Department of Pediatric Neurology, Yüzüncü Yil University Faculty of Medicine, Van, Turkey; 4 Department of Neonatology, Yüzüncü Yil University Faculty of Medicine, Van, Turkey

**Keywords:** Child, mercury, poisoning

## Abstract

Mercury exists as organic inorganic and elementary forms in nature and is one of the most toxic metals that are poisonous for human beings. Mercury is commonly used in many different sectors of industry such as in insects formulas, agriculture products, lamps, batteries, paper, dyes, electrical/electronic devices, jewelry, and in dentistry. In this study, two siblings (one a 7-year-old boy and the other a 13 years old girl) are reported who developed chronic mercury poisoning as a result of long-term contact with batteries. Our aim is to emphasize the importance of mercury poisoning that is extremely rarely seen in childhood.

## INTRODUCTION

Mercury exists in organic inorganic and elementary forms in nature and is one of the most toxic metals that are poisonous for human beings. According to the 1997 recordings of the American Association of Poison Control Center, mercury poisoning ranked second among acute heavy metal poisoning cases. Mercury is commonly used in many different sectors in industry such as in insects formulas, agriculture products, lamps, batteries, paper, dyes, electrical/electronic devices, jewelry, and in dentistry. Both forms of mercury are toxic to humans, while the biological effects of toxicity depend on the form of mercury the person is exposed to. Symptoms such as clinical temperature, coughing, chest pain, hemophthisis, insomnia, depression, exanthema, weakness, fatigue, joint-muscle pains, and the signs of resembling nephritic syndrome can be seen in mercury poisoning.[1–4]

In this study, two siblings (one a 7-year-old boy and the other a 13-year-old girl) are reported who developed chronic mercury poisoning as a result of long-term contact with batteries.

## CASE REPORTS

### Case 1

A 7-year-old boy was referred for pain in his legs, neck, and abdomen. We learned that the boy was suffering from abdominal and neck pains since a period of 2.5 months and that since 20 days he also experienced pain in his extremities. The patient had not fever, vomiting or diarrhea, but had reduced appetite since 2 months and had lost 2-3 kg weight. He had a history of measles and chorcoal poisoning 2 month ago. His sister also had abdominal pain for 2 months. No characteristic features were present in the family lineage. The physical examination revealed the followings: body temperature 37°C; heart rate 110/min; respiration rate 28/min; and arterial blood pressure 100/70 mmHg. Body weight was 26 kg and height was 128 cm. The general condition was moderate, however the patient showed fatigue. A diffused sensitivity was present while the extremities were palpated. On examination other systems were found to be normal. Laboratory test results include the following: routine urinary and blood studies are normal, and liver and renal function tests are normal, creatine kinase: 71 IU/L; lactate dehydrogenase: 316 IU/L; antistreptococcal antibody (ASO): 57 TU; C-reactive protein (CRP): <3 mg/dL; ANA: negative; antiDNA: negative; C3: 1.14; Brucella agglutination test: negative; Gruber-Widal agglutination test: negative; thyroid function tests were within the normal limits. Blood lead level: 2.3 ug/dL (N); blood mercury level: 9.3 ug/ dL [Table 1].

**Table 1:** Serum mercury levels of our patients

Period	Case 1	Case 2
At the beginning of the therapy	9.3	13.8
8 week	6.6	12.8
12 week	5.4	8
16 week	-	6.8
20 week	6.5	-
28 week	-	2.5 (discontunied)
29 week	4.5 (discontunied)	-
36 week	3.2	-
37 week	-	2.4
45 week	-	2.7
46 week	3	-
55 week	-	5.4

The patient's history led us to consider chronic mercury intoxication due to clinical features and high mercury levels in blood sample. Therapy with D-penicillamine was initiated. In the second month of the therapy, abdominal pain was diminished and the patient's extremity pain was reduced. At further controls, the patient showed no any complaints and therefore his blood mercury levels were assessed together with 2 months intervals. After receiving D-penicillamine for a period of 8 months, the patient showed no symptoms and is currently being followed-up in our out-patient clinic.

### Case 2

The 13-year-old patient referred to our out patient clinic was diagnosed with chronic mercury intoxication due to symptoms of abdominal pain, extremity pain, and dermal eruptions. The patient's blood mercury level was 13.8 ug/dL. The treatment scheme was initiated with D-penicillamine. Two weeks after therapy, the skin lesions increased in both at the extremities and the gluteal regions and were accompanied with itching and a sensation of burn, pain, and increased temperature; however, no swelling, rash, or increased temperature were observed in the joints. The medical history of the patient displayed no specific features, but his 7-year-old male sibling was also diagnosed with chronic mercury intoxication. Both siblings enjoyed playing with batteries at home.

During the physical examination, body weight was 50.5 kg (50-75 p); and height 154 cm (25-50 p), body temperature was 37.6°C; pulse rate 128/min; and arterial blood pressure was 110/80 mmHg. The general condition of the patients was good, and accompanied with diffused sensitivity which was present during palpating extremities and increased temperature; however, there were no such findings as increased temperature, rash, swelling, or restricted movement at the joints area. Linear erythematosus areas were present and ulcerated lesions which were significant at the both lower extremities at the gluteal.

Laboratory test results are as follows: urinary analysis shows density: 1036; pH: 5; protein: >300; sulfosalicylic acid test: +++; glucose: negative; ketone: trace. Hemoglobin: 14 g/dL; white blood cell count: 8100/mm^3^ ; mean corpuscular volume:81 fl; mean corpuscular hemoglobin: 28 pg; mean corpuscular hemoglobin concentration: 34 g/dL, thrombocytes: 441.000/mm^3^ ; red blood cell distribution: %12.2; erythrocyte sedimentation rate: 17 mm/h; aspartate aminotransferase: 113 IU/L, alanine aminotransferase: 94 IU/L; and lactate dehydrogenase: 1143 IU/L, creatine kinase: 3044 IU/dL; blood lead level: 2.20 ug/dL; blood mercury level: 12.8 ug/dL (normal:0-10) [Table 1]; ASO, CRP, RF, ANA, and antiDNA were negative. C3 and C4 levels were within normal limits. Salmonella and Brucella agglutination tests were negative. Urinary porphobillinogen and amino-levulinic acid values were normal. EMG showed myogenic involvement. The EMG performed 2 months later was assessed to be normal.

D-penicillamine therapy was immediately initiated in the patient who was hospitalized in our clinic, while naproxen and carbamazepine were also administered in order to reduce the diffuse body pain. Dermal eruptions were considered as halogenodermosis. During controls, we determined an increase in the liver enzyme levels accompanied with proteinuria and therefore D-penicillamine therapy was stopped, considering the possibility of D-penicillamine-induced hepatotoxicity and nephrotoxicity. After stopping D-penicillamine, dermal eruptions vanished. The patient was treated with Dimercaprol instead of D-penicillamine for a period of 10 days. During the controls, liver and renal function tests turned out to be normal and then D-penicillamine therapy was re-initiated. At the first control, 3 weeks after the patient was discharged from the hospital, pain in the extremities and head was reduced and abdominal pain was totally eliminated. In the second month of therapy, the patient's complaints totally eliminated. The patient received D-penicillamine for a total of 7 months, and then the blood mercury level was carefully monitored during bi-monthly controls for a period of 12 months.

## DISCUSSION

Mercury is a non-essential element for biological functions. Therefore, humans exposed to mercury may display symptoms of mercury intoxication. Depending on its form in nature, elementary, organic or inorganic forms, and the entrance route of mercury to the human body may be different. Elementary mercury is commonly absorbed totally by the lungs and therefore its absorption by the gastrointestinal tract and skin is not important. However, ionic mercury salts display different rates of absorption by the lungs and gastrointestinal tract while its absorption by skin is scarce. Organic mercury is commonly absorbed totally by the lungs and the gastrointestinal tract while its dermal absorption may differ between a moderate degree of absorption to a higher degree.[1,3,5] Although heavy metal poisonings in children are not seen very often but these poisoning are known to be environmental and public health-related problems.[6]

In both of our patients, we diagnosed chronic mercury intoxication, merely we were unable to know the source of mercury poisoning. Both patients told us that they frequently played with batteries; but there were no particular past activities to lead us to consider heavy metal intoxication such as playing with thermometers, batteries, fluoresan bulbs, insecticide drugs, antiseptic agents, explosives or any past history of consuming high amounts of fish while there were no past history of family members or the patients themselves that were employed in an industrial sector where mercury was abundantly used. Therefore, in the present mercury intoxication case, we considered elementary mercury (battery) as the source of poisoning.

In the first case, pain was present in the extremities, neck, and abdomen. Physical examination revealed sensitivity in the palpation of leg muscles, and the deep tendon reflexes were reduced. In the second case, complaints were similar and were accompanied with diffused sensitivity during palpation of the extremity muscle plus dermal eruption. It was interesting to find similar findings that resembled polyneuropathy. As muscle and joint retention has a great preliminary importance, it is necessary to eliminate this group of patients by means of rheumatologic studies. When studies related to Salmonella and Brucella agglutination tests and rheumatologic testing are found negative, other conditions such as joint-muscle involvement must be considered. The blood lead level in both of our patients, which can be easily detected in heavy metal poisoning which also leads to the polyneuropathy symptoms, was found to be normal, but high blood mercury levels orientated us to think about the possibility of a chronic mercury intoxication. It was quite interesting to see that symptoms resembling a neuropathological condition were present at the beginning of the preliminary plan, while there were no findings related with retention of the multisystem and central nerve system even though mercury poisoning was diagnosed.

All of the workers who were employed in a thermometer manufacturing factory for a period differing between 1 and 40 years showed high mercury levels in their blood and urine, but only some of the workers demonstrated clinical central and peripheral nerve system symptoms, whereas according to MRI, 88% of the workers had sub-clinical neuropathies. However, no relationship was found between the period of exposure to mercury and the values of blood and urine mercury levels and neuropathies. An electromyelography (EMG) was carried out at the second patient of our cases and a slight myogenic retention was determined. At the second month of therapy, the control EMG was found to be normal.

In a study performed by Chu *et al*, 3 months after the administration of a drug that contains mercuric sulfate, a table of symptoms related to polyneuropathy developed and EMG displayed motor and sensorial involvement while axonal degeneration was also determined during sural nerve biopsy; therefore, it was reported that exposure to inorganic mercury would lead to intensive axonal sensory--motor polyneuropathies in human beings.[7,8]

In cases with a central nerve system involvement dependent on mercury intoxication in the literature, interpretation is performed according to these tests and neuro-functional assessments were carried out in the present cases. There was no need to perform neuro-function tests on our patients as they all lacked central nerve system symptoms. However, as many cases can be found in the literature that involves chronic mercury intoxication which shows progress with papular and papulovesicular, purulent or non-purulent lesions, in our second case dermal eruptions were present, and this dermal finding was considered as a drug reaction, because dermal findings had initiated with the administration of D-penicillamine and complaints dramatically improved after this drug was interrupted.[3,4,9] In mercury poisoning, the blood mercury level may be misleading, therefore to obtain a reliable diagnosis, it would be appropriate to determine the mercury level both in urine and blood after the administration of drugs that may increase mercury discharge from the body. In published studies, it was found that the mercury level of hair demonstrated strong relationship with clinical findings.[1,2,4,10] In both patients, diagnosis was performed according to the medical story of the patients, clinical findings, and high blood mercury levels. Additionally, our diagnosis was confirmed after a decrease occurred in the blood mercury level as a response to therapy.

Consequently, mercury intoxication must be considered in patients who referred to symptoms of abdominal pain, extremity pain.
